# Cancer metastasis chemoprevention prevents circulating tumour cells from germination

**DOI:** 10.1038/s41392-022-01174-w

**Published:** 2022-10-02

**Authors:** Xiaodong Xie, Yumei Li, Shu Lian, Yusheng Lu, Lee Jia

**Affiliations:** 1 College of Materials and Chemical Engineering, Minjiang University, Fuzhou, Fujian 350108 China; 2 School of Basic Medicine, Gannan Medical University, Ganzhou, Jiangxi 341000 China; 3 Cancer Metastasis Alert and Prevention Center, College of Chemistry, Fujian Provincial Key Laboratory of Cancer Metastasis Chemoprevention and Chemotherapy, Fuzhou University, Fuzhou, Fujian 350116 China

**Keywords:** Cancer therapy, Metastasis

## Abstract

The war against cancer traces back to the signature event half-a-century ago when the US National Cancer Act was signed into law. The cancer crusade costs trillions with disappointing returns, teasing the possibility of a new breakthrough. Cure for cancer post-metastases still seems tantalisingly out of reach. Once metastasized, cancer-related death is extremely difficult, if not impossible, to be reversed. Here we present cancer pre-metastasis chemoprevention strategy that can prevent circulating tumour cells (CTCs) from initiating metastases safely and effectively, and is disparate from the traditional cancer chemotherapy and cancer chemoprevention. Deep learning of the biology of CTCs and their disseminating organotropism, complexity of their adhesion to endothelial niche reveals that if the adhesion of CTCs to their metastasis niche (the first and the most important part in cancer metastatic cascade) can be pharmaceutically interrupted, the lethal metastatic cascade could be prevented from getting initiated. We analyse the key inflammatory and adhesive factors contributing to CTC adhesion/germination, provide pharmacological fundamentals for abortifacients to intervene CTC adhesion to the distant metastasis sites. The adhesion/inhibition ratio (AIR) is defined for selecting the best cancer metastasis chemopreventive candidates. The successful development of such new therapeutic modalities for cancer metastasis chemoprevention has great potential to revolutionise the current ineffective post-metastasis treatments.

## Introduction

Currently, much advance in cancer care, prevention, and research has been made.^1^ The fatal blood-forming malignancies, such as chronic myeloid leukaemia (CML), now show ~90% overall survival.^2^ Targeted anticancer drugs produce significant short-term remissions of some hard-to-treat cancers, like metastatic melanoma and non-small-cell lung cancer, and have hence transformed the care for patients with cancer.^1^ However, the goal of “Eliminating the suffering and death due to cancer”^3^ is still far from our reach.

**Fig. 1:**
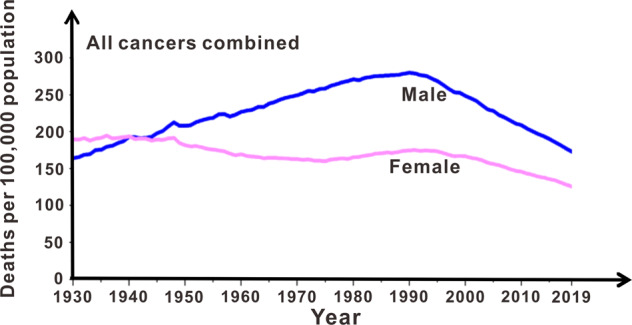
Eighty-nine-year death rate of all cancers combined of American men and women^4^

The latest global cancer data showed that in 2020, cancer burden increased to more than 19.3 million new cases, and 10.0 million cancer deaths of both sexes and all ages combined,^4^ indicating that no significant achievements have been made in reducing cancer death. Compared to the theranostic victories over other devastating diseases, we are sceptical about the 50-year-old strategies and directions in cancer post-metastasis treatments. There has no satisfactory decline in the number of deaths from cancer in the past 89 years (Fig. 1). In the early 1980s, people with HIV were unlikely to live longer than a few years, whereas currently there are 31 anti-HIV drugs approved by the US FDA, which convert HIV infection into a manageable disease, and sometimes even suppress the viruses to undetectable levels.^2^ Here we present cancer pre-metastasis chemoprevention strategy that can prevent circulating tumour cells (CTCs) from initiating metastases safely and effectively, and is disparate from the traditional cancer chemotherapy and chemoprevention. Researchers should make cancer metastasis chemoprevention a clinical reality.^5^

## The disappointing war on cancer

Over the past half a century, more than 600,000 compounds have been screened and tested for their anticancer activities, of which only ~40 drugs can be routinely used in clinics.^6^ Development of anticancer drugs has been shifted to the progress of cytotoxic and cytostatic drugs, often resulting in frustrating results due to drug toxicities and side effects, inter- and intra-tumour heterogeneity, cancer drug resistance,^7^ and cancer microenvironmental reprogramming.^8,9^ More disappointing is the fact that these anticancer drugs cannot reverse the death of patients after cancer metastases. As a result, 17% of American patients with stage IV colorectal cancer have given up on the hopeless treatments.^10^ Recent understanding of the importance of cancer microenvironment is revealing immunological dynamics between cancer cells and the host.^9^ Strategies for developing adoptive cell therapy, therapeutic vaccines, immune checkpoint inhibitors (e.g. PD-1 and PD-L1) have provided alternative therapies.^11,12^ However, the current immunotherapy and precision medicine benefit only a few patients whose cancer signature molecules can match the effective drug(s). Sadly, the immune checkpoint inhibitors were found to produce serious side effects.^13–15^

Considering the high side effects, and low efficacy of traditional cancer post-metastasis therapies in the past half a century,^16^ cancer therapies urgently need new ideas and new directions in the era of AI and big data with sea information. The new direction should be revolutionarily different from what we have been doing till date. Otherwise, we will basically be following similar strategies in the remaining half of the century. Here, we present the novel strategy of cancer metastasis chemoprevention.

## Cancer metastasis chemoprevention

**Fig. 2:**
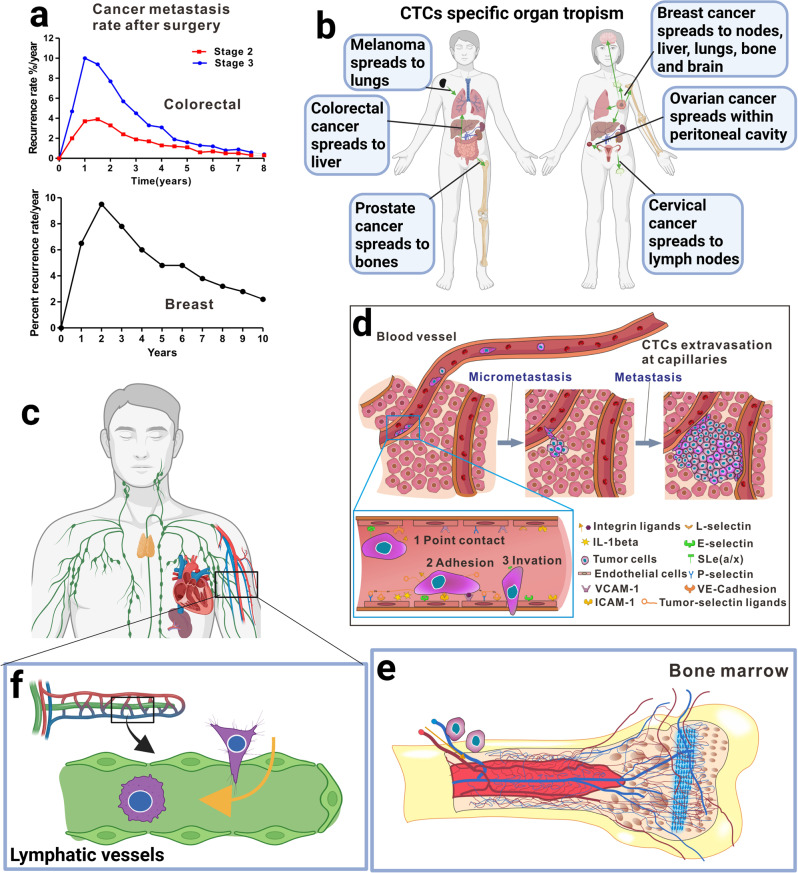
The revolutionary and innovative cancer metastasis chemoprevention strategy for preventing cancer cell dissemination from starting. **a** Local tumours can be precisely removed, however, cancer metastases often occur years after surgery. The data are summarized and redrawn from trials of >20,000 colorectal cancer patients (upper panel) and 37,000 of breast cancer patients (lower panel). **b** Each type of cancer has its own specific metastasis organotropism that helps us design metastasis chemopreventive agents based on different cancer type. **c** CTCs travel through blood and lymphatic systems, and disseminate to the distant metastasis tissues via capillary extravasation. **d** Adhesion of CTCs to capillary endothelial cells is the first and the most important step for CTCs in niches to initiate the micrometastases and metastasis over the body. The inset shows how CTCs in the niches are affected by many inflammatory factors and adhesion-related molecules to complete their adhesion/invasion processes. **e** CTCs stay dormant in the bone marrow before activated. **f** Some CTCs stay hidden in lymph nodes. Created with biorender

Contrary to the situation 50 years ago, most patients currently do not die of primary cancer, but rather of cancer metastases that occurs 1–5 years after surgical resection of primary cancer.^17^ This clinical result suggested that the local primary cancer is no longer life-threatening. Using state-of-the-art equipment, including magnetic resonance, computed tomography, nuclear medicine, sonography, interventional radiology, and fusion modalities, as well as the commercially available types of imaging probes and new diagnostic contrast agents, local pre-metastasis tumours can now be precisely removed. However, 1–5 years after surgery, 30–70% (depending on the types of cancer, stages, and other factors) of cancer survivors show metastases (Fig. 2a). Among colorectal cancer patients undergoing a curative resection (R0), approximately half of them die of cancer metastasis within 5 years. Even patients with lymph node-negative (N0) cancer have the metastasis rate as high as 30%. Even more worse progonsis was exiseted in lung cancer, with 60% of R0 and 40% of N0 patients dying of metastasis. In breast cancer, the metastasis rate is 25–30%, and in prostate cancer, it is 15–50%.^18^ Metastasis can actually occur many years after surgical resection of primary cancer. Till date, cancer metastasis remains difficult to cure.^1,16,19^ Facing the great difficulty to cure the cancer post-metastasis cases, we hypothesised that it may be easier to prevent cancer metastasis before it starts than to treat it after metastases. We therefore coined the term cancer metastasis chemoprevention.

**Fig. 3:**
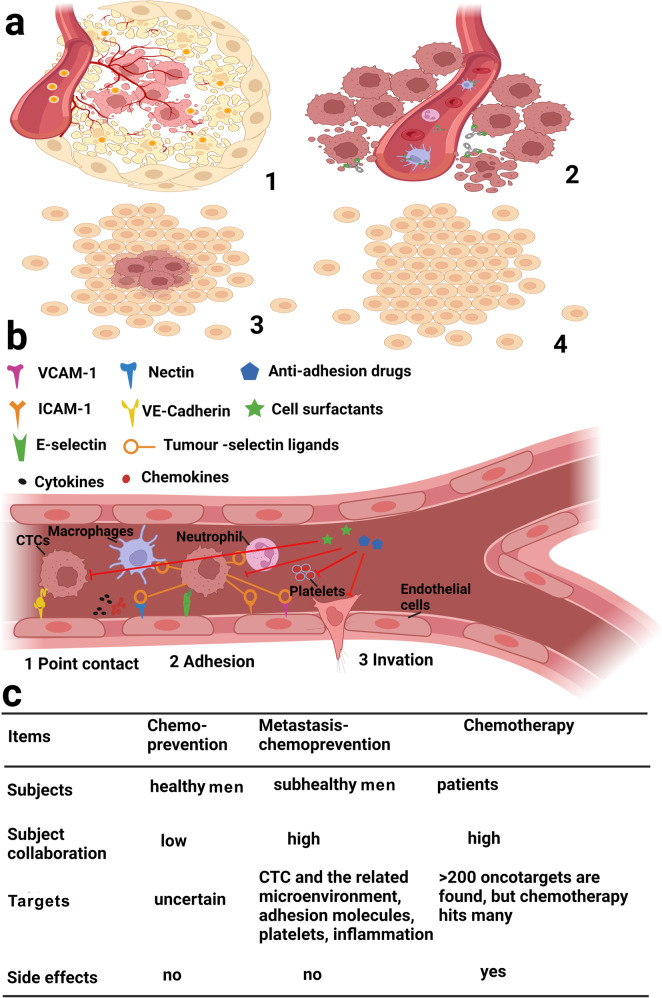
Differences between traditional chemoprevention or chemotherapy and cancer metastasis chemoprevention. **a**1 Chemotherapy often destroy both tumours and normal tissues; **a**2 unlike a ballistic missile that is guided by radar or GPS to destroy the target eternally, the target drugs (green) often are off-target, and cannot destroy the cancer forever; moreover, the blockbuster target drugs hit not only one super-target, but also other interconnected targets; **a**3 cancer metastasis chemoprevention can start immediately after the tumour is confirmed. A tumour can be surgically removed, **a**4 followed by using cancer metastasis chemopreventive agents to prevent CTCs from seeding. **b** Cell surfactants or anti-adhesive agents inhibit inflammatory factors (cytokines, NF-kB), cell adhesive molecules, chemokines, E-cadherin, vimentin and platelets, thereby, preventing these factors from sparking adhesion/invasion of CTCs. **c** Significant differences in subjects’ collaborations, drug targets, side effects among traditional chemoprevention, chemotherapy and cancer metastasis chemoprevention. Created with biorender

**Fig. 4:**
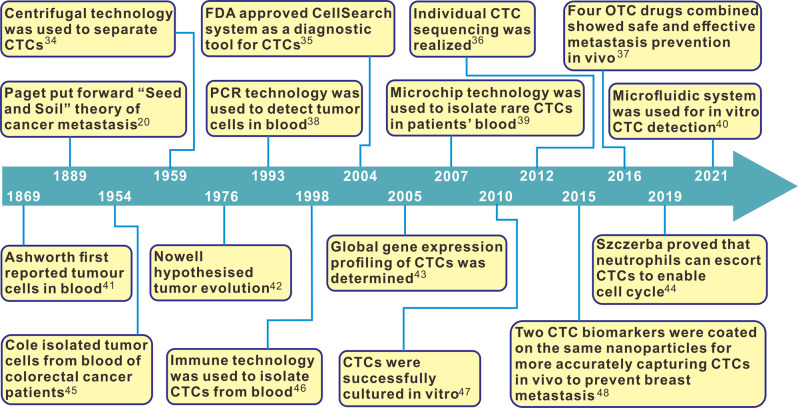
The most influential events in history contributing to discovery and characterization of CTCs

Cancer metastasis chemoprevention is characterized as the utilization of natural, synthetic, and biological agents or their combination to safely interrupt adhesion/invasion of CTCs onto the microvascular endothelium of metastatic tissues, and thereby prevent blood CTCs and their relevant molecules from germinating into metastatic tissues (Figs. 2d, 3a, b). The fundamental difference in cellular mechanism between cancer metastasis chemoprevention and the traditional cancer chemoprevention or cancer chemotherapy is that the cancer metastasis chemoprevention does not intend to prevent carcinogenesis, as in cancer chemoprevention, nor to kill cancer cells, as in cancer chemotherapy. Instead, the cancer metastasis chemoprevention focuses on preventing the metastasis from happening immediately after the primary pre-metastasis cancer (at or earlier than the cancer stage T1–2N1–2M0) is diagnosed and confirmed with or without further treatments. A pre-metastasis tumour can be surgically removed, followed by using cancer metastasis chemopreventive agents to prevent CTCs from seeding (Fig. 3a3, a4). There are many factors presented in the CTCs microenvironment niches, which could favour or enhance adhesion/invasion of CTCs to endothelium. These factors include inflammatory factors (cytokines, NF-kB), cell adhesive molecules, chemokines, E-cadherin, vimentin and platelets. Any cancer metastasis chemopreventive agents or their combination that can inhibit the activity of these factors should be able to prevent sparking of adhesion/invasion of CTCs to their microenvironment niches. Sir James Paget had hypothesised (Fig. 4) that “even a well-defined virus can produce its appropriate disease only in some exactly appropriate place or texture”.^20^ If grass (the microenvironment niches) is well maintained, weed (the CTCs) cannot find a space on soil to seed and germinate.

There are three significant differences between cancer metastasis chemoprevention and the traditional cancer chemoprevention or chemotherapy (Fig. 3c): (1) Subject classification and subject’s motivation to collaborate with the treatments: in the case of cancer metastasis chemoprevention, the subjects are subhealthy persons who are diagnosed with pre-metastasis cancer and are told that the cancer metastasis is life-threatening, and they will highly collaborate with cancer metastasis chemoprevention. The same degree of collaboration exists in cancer chemotherapy where the subjects are cancer patients who are fear of death caused by cancer, and will highly collaborate with chemotherapy. However, in cancer chemoprevention, the subjects are healthy persons, they may sometimes ignore chemopreventive agents or dietary-derived materials, and regard these agents unnecessary. (2) Drug target classification: in the case of cancer metastasis chemoprevention, the targets are CTCs and their related adhesion molecules, inflammatory factors in the niches. In the case of cancer chemoprevention, the targets are uncertain. Whereas, in the case of cancer chemotherapy, there are more than 200 oncotargets found, and these oncotargets are reportedly interconnected, and therefore, precise-targeting is very difficult, if not impossible. (3) Side effects of treatments: in the case of cancer metastasis chemoprevention, the side effects can be controlled as low as cancer chemoprevention. Whereas, chemotherapy often produces intolerable side effects.

Chemoprevention of cancer metastasis is disparate from existing cancer chemotherapy and chemoprevention (Fig. 3). The traditional cancer chemoprevention is defined as the use of natural, synthetic or biological agents to reverse, restrain or prevent either the initial phases of carcinogenesis or the progression of premalignant cells to invasive disease.^21^ The process of tumour initiation, promotion, and progression can be perturbed in cancer chemoprevention. There are a number of potential mechanisms that have been proposed to classify agents according to the effects they have on different stages of carcinogenesis. Many cancer chemopreventive agents are dietary-derived and aim at multiple targets. These agents often contain (1) dietary-derived materials such as β-carotene, folic acid, retinol and retinoids, vitamin E, vitamin C, multivitamin supplements, calcium and selenium; and (2) agents targeting towards metabolic and hormonal pathways including statins, oestrogen and antagonists, 5α-reductase inhibitors.

The traditional cancer chemopreventive agents are supposed to prevent carcinogenesis, reduce the possibility of biological damage and mutations which contribute not only to cancer initiation but also progressive genomic instability and overall neoplastic transformation. Cancer chemoprotection may be achieved as a consequence of decreased cellular uptake and metabolic activation of pro-carcinogens and/or enhanced detoxification of reactive electrophiles and free radical scavenging, as well as induction of repair pathways.^22,23^ Downregulation of chronic inflammatory responses and the production of reactive oxygen and nitrogen species may also contribute to the prevention of cancer initiation. Other protective processes include regulation of DNA methyltransferases to prevent or reverse the hypermethylation-induced inactivation of tumour suppressor genes. Among the various effects of cancer chemopreventive compounds on epigenetic mechanisms of cancer, inhibition of histone deacetylases has also been demonstrated.^24^ Once primary tumour has formed, cancer chemopreventive compounds should influence the progression of initiated cells. This activity is primarily mediated by inhibiting signal transduction pathways, for example, such as by targeting nuclear factor NF-kB to disturb the effects of tumour promoters,^25^ which would otherwise lead to cell proliferation.

Clinical use of the traditional cancer chemopreventive agents relates to the using of drugs to healthy people or to people at risk for disease despite having no obvious symptoms. Cases may contain the administration of drugs like oltipraz, which lead phase I or II enzymes to adjust metabolic effects of carcinogens on exposed people. Traditional cancer chemoprevention also relates to identifying people with precancerous lesions and using of drugs to avoid development to invasive cancer. This would include the use of non-steroidal anti-inflammatory drugs (NSAIDs) in patients with colorectal adenomas. However, the chronic administration of cancer chemopreventive agents without specific disease targets and/or disease biomarkers often results in failures of the projects, or inconclusion.^26^

The traditional cancer chemoprevention is almost a failure although a few successes were shown.^26^ Some cancer chemopreventive studies showed null prevention,^27^ others showed opposite results.^28^ The SELECT trial is a good example of the failure of the traditional cancer chemoprevention, in which 35,533 men from 427 participating sites across the United States, Canada, and Puerto Rico were randomly assigned to 4 groups (selenium, vitamin E, selenium+ vitamin E, and placebo) in a double-blind fashion between 22 August 2001 and 24 June 2004, and results of the trial suggested that selenium and vitamin E, in combination or separately, failed to prevent prostate, lung, and colorectal cancers,^29^ and showed a statistically non-significant increase in prostate cancer risk with vitamin E. To further determine if the prolonged administration of vitamin E and selenium could have chemopreventive effect on risk of prostate cancer in relatively healthy men, the continuous study planned a follow-up of 7–12 years, and finally concluded that dietary supplementation with vitamin E actually significantly increased the risk of prostate cancer among healthy men.^30^

Possible reasons underlying the lack of safe and effective metastasis chemopreventive drugs are as follows: (1) Over the past 50 years, as a result of various subject specializations, there has been limited communication, understanding, and collaboration across molecular geneticists, oncologists, immunologists, pharmaceutical scientists, and clinicians working in the field of cancer. (2) The animal models (Table 1) used for preclinical anticancer drug screening do not match clinical reality. Till date, a criterion for choosing an anticancer drug as a candidate for subsequent tests is whether it can kill the selected types of cancer cells in vitro at a concentration lower than micromolar level, and shrink the subcutaneously implanted tumour grown from cancer cells in vivo.^7,8,31^ In the clinics, however, surgical removal of a local tumour is always the first choice of cancer therapy, unless metastasis has already set in. For local tumours, treatment to shrink the tumour should be a second-line choice, unless surgery is inconvenient for the particular tumour. The sensitivity and specificity of clinical tumours to an anticancer drug is usually not high enough for using an anticancer drug only, and off-target and related serious side effects are common. (3) Previously, cancer metastasis was an ignored topic. Examining the NCI grant proposals since 1972 revealed that <0.5% of study proposals had focused primarily on metastasis. Out of ~8900 NCI grant proposals awarded in 2003, 92% did not even mention the word “metastasis”.^16^ (4) As the science of cancer prevention improves, it must be remembered that effective and efficient preventive services do not help if they are not used. It is difficult to motivate practitioners and patients to implement preventive services. The topic of cancer chemoprevention was not raised until recently exclusively due to the absence of such a health care insurance. Only recently, some European companies have started covering a number of compounds with proven cancer chemoprevention ability, as it has been calculated that it is prudent and cheaper to prevent cancer than to treat it.^32^ Nevertheless, the safe and effective cancer metastasis chemoprevention, focusing on preventing cancer metastatic cascade from getting triggered is a much-needed and revolutionary strategy.

**Table 1:** Pros and cons of the various mouse tumour models

Animal models	Tumour types & homology to humans	Tumour growth time & take rate	Host immune system	Preclinical management	Others
Transplantable murine tumours grown in syngeneic hosts	Murine tumours (P388, LI1210, Colon 38, B16, M5076, Lewis lung); poor human homology	<2 days; high take rate	Intact, competent	Easy	Metastasis suitable
Xenografts of human tumours grown in immunodeficient murine (NCR-nu/nu; SCID; RH-Foxn1rnu)	All human tumours, if available; good human homology; original human tumour cells may not be uniform after many passages	From 1 to 10 weeks; melanoma and colon grow fastest, breast and lymphoreticular grow slow; matrigel helps	Deficient in CD4^+^ and CD8^+^ T cells; SCID lacks B and T cells; accept allografts and xenografts; possible T cells grow when murine age	Ease to monitor s.c. tumour growth	*Foxn1nu* gene is mutated; Used for AIDS and leprosy
Orthotopic metastatic models of human tumours (OMMHU)	Colon, melanoma, breast, kidney	Weeks, but demand surgical skills	Can be intact	Difficult to monitor tumour growth*	
Genetically engineered mouse (GEM)	Very homologous to humans	Long, months–1 year; heterogeneity in tumour growth and latency	Competent	Individual management	Patent rights (2016)
Orthotopic patient-derived xenografts in immunocompromised mice	Paediatric solid tumour (including epithelioid sarcoma, Ewing sarcoma, osteosarcoma, retinoblastoma, rhabdomyosarcoma, Wilms tumour, etc.); original human tumour cells	Long, 1.25–11.75 months; overall engraftment efficiency was 45%	SCID	Difficulty varies with the type of cancer	^52^

## CTC biology and detection technology

CTCs are considered the root cause of cancer metastasis and the resulting cancer deaths.^33^ Figure 4 shows the most important events in history regarding research and characterization of CTCs.^20,34–48^ CTCs are shed from primary or secondary tumours after degradation of the extracellular matrix (ECM), and intravasation into blood and lymphatic circulation; they are present at low concentrations with normal blood components in the peripheral lymph system and blood system of patients with cancer and of asymptomatic cancer survivors (Fig. 2). CTCs that get arrested in the vasculature of bone marrow can disseminate to distant metastatic organs through blood circulation system, or remain dormant in the bone marrow. The three possible fates of CTCs are as follows: majority of CTCs die due to blood shear stress, immune attack, and anoikis effect, some enter a state of dormancy in the bone marrow, and others form micrometastasis after successful adhesion/invasion through endothelial capillaries (Fig. 2c–e).^18^

Dynamic polarization of tumour cells is essential for metastasis. CTCs have to survive and readjust to different microenvironments, such as lymph- or blood circulation, where they develop a high degree of plasticity that renders them adaptable to varying conditions,^49^ as sowing the ‘seeds’ of metastasis requires the ‘soil’ at distant metastatic sites to encourage the outgrowth of incoming CTCs.^50^ One defining characteristic of the metastatic embedding is the transition of CTCs between different polarized phenotypes, ranging from differentiated epithelial polarity to migratory front-rear polarity. Single-cell polarity adopted by CTCs in blood constitutes a mode of polarization of the cell cortex that is different from the intracellular polarization machinery, which distinguishes single CTC polarity from other types of polarity identified so far, and contributes to CTC metastasis. While the role of cell polarization during dedifferentiation and migration is well established, polarization of CTCs during phases of detachment has not been investigated. Lorentzen et al. identified and characterized a type of single-cell polarization in liquid phase.^51^ The single-cell polarity is an inherent feature of cells from different tumour entities. Functionally, the single-cell polarity is directly involved in early CTC attachment, thereby affecting their adhesion, transmigration and metastasis.

The metastatic capacity of CTCs in vivo correlates with the extent of single-cell polarization. Manipulating the polarity regulators or generic depolarization may affect transmigration and metastasis of CTCs in vitro and in vivo, and thereby contributing to a part of targeted therapy.^53^

In fact, only a small subset of CTCs is capable of successful metastasis, therefore these cells are termed circulating cancer stem cells (cCTCs^54^). cCTCs bear metastasis-initiating capabilities based on their stemness properties and invasiveness.^55^ As compared to non-tumorigenic/metastatic bulk CTCs, cCTCs may not only be capable of evading from the primary tumour, but also escape from immune surveillance, survive in the circulating blood and subsequently form metastases in distant organs. Thus, cCTCs represent a subset of exclusively tumorigenic cancer stem cells characterized by their invasive characteristics and are potential therapeutic targets for preventing disease progression. cCTCs are critical for the patients’ clinical outcome.^56^

Recent availability of organ-on-chip models and biobanks provides us with a new window to understand the cancer metastasis.^57^ In biobanks, patient cases and related information are stored anonymously and marked with a PIN code that is generated based on the individual biological characteristics to uniquely distinguish the sample depositors.^58,59^ Cancer organoid culture platforms can capture the heterogeneity and pharmacotypic signatures of the parental tumours. Clinical trials have been performed using patient-derived organoids as a tool for personalized medical decisions to predict patients’ responses to therapeutic regimens and potentially improve treatment outcomes.^60^ Living organoid biobanks encompassing several cancer types have been established, providing a representative collection of well-characterized models that will facilitate drug development. Kopper et al. have established 56 organoid lines from 32 ovarian cancer patients, which represented all main subtypes of ovarian cancer.^61^ These ovarian cancer organoids recapitulated histological and genomic properties of the relate lesion from which they were originated, illustrating intra- and interpatient heterogeneity, and can be modified genetically. The in vitro organoid-on-a-chip, and more complex multi-organoid body-on-a-chip platforms mimic human physiology and cancer pathology more precisely than traditional 2D cultures,^62,63^ and can be used for drug-screening assays and capture different tumour subtype responses to the gold standard chemotherapy, including acquisition of drug resistance in recurrent disease. The cancer organoids can be xenografted, enabling in vivo drug-sensitivity assays,^61^ and also used for CTC-related research.^64^

It is important to note, however, that cancer stem cells do not necessarily represent bona fide stem cells nor do they necessarily arise from tissue stem cells, but rather cancer stem cells have acquired certain traits of stem cells allowing them to indefinitely self-renew and give rise to their respective differentiated progenies. Several of the most commonly used cancer stem cell biomarkers are CD44, CD24, CD133, CD166, and ALDH1. ATP-Binding Cassette Transporters (ABCG2, ABCB5), EpCAM, CXCR4, nestin and LRCs have also been utilized for the identification of cancer stem cells.^65–67^ It is not surprising that CTCs share some biomarkers with cCTCs because they come from the same cell origin.

Till date, ex vivo cultures of various CTCs have been completed, including those of breast,^68^ prostate,^69^ pancreatic^70^ and colon CTCs,^71^ and they have been well characterised in vitro.^72,73^ A wide variety of CTC enrichment and detection methods has been developed over the past decade.^74–77^ Clinical detection of CTCs has become a new biological industry. The detection methods range from cell size-based separation,^78^ nucleic acid-based assays using reverse transcription–polymerase chain reaction,^79^ immunocytochemistry assays,^80^ and the recent ultrahigh-throughput magnetic sorting of large blood volumes for CTC isolation^81^ to the use of immunomagnetic beads conjugated with an antibody to epithelial cell adhesion molecule (EpCAM),^39,82^ a commonly expressed epithelial cell surface marker.^83–85^ One of the most commonly used methods for CTC detection is to differentiate CTCs via surface biomarkers that are not expressed in the normal haematologic cells. The surface biomarkers include EpCAM,^86^ human epidermal growth factor receptor,^87^ prostate-specific antigen,^88^ epidermal growth factor receptor,^89^ and carcinoma embryonic antigen.^90^ An effective approach for isolating CTCs from blood samples of patients with pancreatic, breast, and lung cancers can separate CTCs from patient’s blood at a low concentration (3–5 CTCs per ml blood) using functionalised graphene oxide nanosheets on a patterned gold surface.^91^ The CellSearch system (Veridex) uses ferrofluids loaded with an EpCAM antibody to capture CTCs, which are subsequently visualised by staining with a cocktail of antibodies against cytoplasmic epithelial cytokeratins (CKs).^92,93^ Some cancer patients experience recurrence of tumours after a curative-intent treatment. The recurrence is clinically named as minimal residual disease (MRD) caused by the persistence of residual tumour cells. Although MRD cannot be identified by standard radiological examination or clinical evaluation, alterations in tumour-specific biomarkers in the blood can be used to indicate the presence of MRD. Liquid biopsies for CTCs, circulating tumour DNA (ctDNA), or tumour-specific microRNA have been tested for detecting MRD.^94^ Although numerous methods have been developed for the enrichment and detection of CTCs, none has yet reached the ‘gold’ standard and no cut-off criteria have been verified and accepted. Since EpCAM-based enrichment of CTCs has several advantages, it is one of the most commonly used high-throughput method. However, CTCs are heterogeneous, consisting of epithelial tumour cells, epithelial-to-mesenchymal transition (EMT) cells, irreversible EMT( + ) tumour cells, and circulating tumour stem cells (CTSCs). The EpCAM-based approach may not detect CTCs expressing low levels of EpCAM and non-epithelial phenotypes, such as CTSCs and those that have undergone EMT and no longer express EpCAM. More accurate CTC detection methods should be developed to simultaneously measure both epithelial biomarkers, such as CD176, EGFR, HER2, Muc1, CD26, CD44, CD133, CXCR4, and EMT+ biomarkers, like N-cadherin, O-cadherin, vimentin and fibronectin.^95^

With the development of such separation and culture technologies, biological characterisations of cultured CTCs have been possible.^68–73^ CTCs can be biologically stable for more than 2 years; they are CD45-negative and non-adherent cells, and have an intermediate EMT phenotype, showing bone-marrow origin and sharing the main features of original primary tumour and lymph node metastasis with epithelial properties and stem-cell characteristics; they are tumorigenic in SCID mice, and can induce in vitro angiogenesis.^96^ Nonetheless, specifically targeting blood CTCs without producing any side effects remains difficult, if not impossible, to achieve.^97^

## Metastasis starts with adhesion

Clinical studies have demonstrated that the number of CTCs in blood is a robust independent prognostic factor indicating whether patients, after surgical removal of tumours, are at early pre-metastatic or late post-metastatic stage.^73,98^ In the early pre-metastatic stage, pathologists can hardly find CTCs in the lymph nodes proximal to the removed tumour (Fig. 2f). To colonise distant organs, CTCs must overcome many obstacles including EMT^99^ that is also crucial for embryogenesis and wound healing. During EMT, cell–cell and cell-extracellular matrix interactions are remodelled, which leads to the detachment of epithelial cells from each other and from the underlying basement membrane, the detached epithelial cells subsequently intravasate to blood as CTCs (Fig. 2d).

Clinical observations have suggested that different types of cancer CTCs metastasize to their specific organs, a process known as “organotropism” (Fig. 2b).^100,101^ Intrinsic properties of CTCs and their interaction with unique features of host organs together determine organ-specific metastatic destination.^102^ The metastatic organotropism exhibited by CTCs during metastases is a multi-step process, regulated by the disseminating and surviving CTCs and their secreted microvesicles,^102,103^ as well as the endothelial suitability of the metastatic niches to favour CTC seeding. To successfully metastasise to distant organs, CTCs in circulation must evade immune attack, survive shear stress, adapt to microenvironment, and quickly adhere to the endothelial membrane with the help of favourable platelets, inflammatory factors, and cell adhesion molecules, followed by invasion and infiltration, and extravasation. Although the obstacles make metastasis a highly inefficient process, resulting in the survival of only <0.1% of CTCs, we hypothesised that inhibition of the first and the most important step, i.e., adhesion of CTCs to the endothelial membrane, can make metastasis impossible. We have tested and verified the hypothesis with five safe and effective metastasis chemopreventive agents or their combinations.^37,104–114^ Fifty-year clinical data have demonstrated that once metastasis is established, treatments fail to provide durable responses,^1,19,115^ and metastasis-related deaths cannot be reversed, no matter of how many anticancer drugs are combined.^116^

Heterocellular adhesion under shear stress is a crucial issue in some pathogenesis, such as immune response, CTC/endothelium adhesion to initiate micrometastasis, ox-LDL-induced monocyte/endothelium adhesion to start atherosclerosis micronucleus, and bacterial infection.^117^ Adhesion of a floating cell to the extracellular matrix, or CTCs to capillary endothelium, or an embryo to endometrium involves complex couplings between cell biochemistry, structural mechanics, and surface bonding. The interactions are dynamic and act through association and dissociation of bonds between large cells, bacteria, and their support matrix at a rate that changes considerably under different microenvironments.^118^ Therefore, how a CTC can quickly develop a steady adhesion remains unknown. Nonetheless, from the point of cancer metastasis chemoprevention, it would be important to explore the complexity of CTC adhesion to the surface of capillary endothelium, including how cell signalling processes strengthen the adhesion and how forces applied to cell-surface bonds act on intracellular sites to catalyse chemical processes or switch molecular interactions on and off, and in particular, how the adhesion and spreading processes can be pharmacologically controlled.

Theoretically, there are two kinds of heterocellular adhesion,^119^ namely (1) the traditional receptor-ligand bond (so-called slip bond),^120^ which describes the binding between receptors and ligand molecules, its lifetime decreasing with tensile force applied to the bond, and (2) adhesion of leucocytes to selectin–ligand bond (so-called catch bond),^121^ which describes rolling adhesion of cells in a hydrodynamic environment, its lifetime increasing with applied tensile force. Increasing levels of shear rate stabilise leucocytes rolling under flow. The mechanism governing the kinetics of rolling adhesion of CTCs to endothelial membrane under shear stress is not yet clear.

The size of cell adhesion and spread determines whether individual floating cells would survive or die.^122^ The size of cell adhesive geometric islands has been demonstrated to switch mammalian capillary endothelial cells from growth (if 100% attached) to apoptosis (if not attached). Cell spread can vary while maintaining the total cell-matrix contact area constant by changing the space between multiple focal adhesion-sized islands. Microenvironmental geometric control of cell viability may represent a fundamental mechanism for adhesion-invasion-survival of a floating cell under shear stress and immune attack. For example, activation of FAK contributes to a variety of biological functions, such as cell adhesion and spreading, cell migration, cell survival and apoptosis, and cell cycle regulation and proliferation.^104^

Anoikis is a term used for describing cell apoptosis that results from loss of cell-matrix interaction. Frisch and Francis had created the term ‘anoikis’, a Greek word to explain a special kind of cell death.^123^ In the last couple of years, many researches have uncovered that the loss of connection to ECM, i.e., non-adhesiveness, can contribute to a unique variety of cellular and molecular changes that ultimately facilitate the apoptosis of ECM-detached cells.^124,125^ The cellular changes reduce the survival opportunities of ECM-detached cells.^126^ Resistance to anoikis may allow the survival of CTCs in systemic circulation, thereby facilitating their metastases to distant organs.^127^

Based on our understanding of the mechanism of CTC-induced metastasis, we innovatively supposed that the adhesion/invasion of CTCs to distant metastatic tissues is the key starting step of cancer metastatic cascade. This starting reaction requires the participation of a variety of inflammatory factors, adhesion molecules, integrins, and platelets (Table 2), and the reaction can be completed only under the most appropriate spatiotemporal conditions, leading to systemic cancer metastases. If a drug can interfere with the adhesion of CTC to capillary endothelial cells, which is a significant starting step for metastasis, it may effectively prevent CTC-induced metastases before and/or after tumour removal.

**Table 2:** Components in CTC microenvironments that affect heterotypic adhesion between CTCs and endothelial cells

Names	Components and structure	Functions	Ref.
Nectins	Immunoglobulin-like cell adhesion molecules (CAMs).	Homophilically and heterophilically interact in trans with each other or with other CAMs to form cell–cell adhesions. Nectins bind protein afadin.	^195^
ICAM	Type I transmembrane glycoproteins, contain 2–9 immunoglobulin-like C2-type domains. ICAM family consists of five members, designated as ICAM-1 to ICAM-5.	Functions as intercellular adhesion molecules, recruit activated leucocytes by binding to leucocyte integrins CD11/CD18, such as LFA-1, during inflammation and in immune responses.	^141,196^
VCAM	A 90-kDa glycoprotein predominantly expressed in endothelial cells upon activation by ROS and cytokines (TNFα, IL-1).	VCAM-1 acts as a cell adhesion molecule by directly interacting with α4β1 integrin expressed on leucocytes via VCAM-1’s Ig-like domains 1 and 4 within the extracellular domain. VCAM-1 plays a key role in connecting leucocytes with activated endothelial cells.	^142^
Integrins	An obligate heterodimeric cell surface receptor, composed of α and β subunits, possessing a transmembrane structure. At least 24 distinct integrin heterodimers are formed by the combination of 18 α-subunits and 8 β-subunits.	Facilitate the attachment of cells to the ECM and mediate signal transduction from ECM to the cells.	^131^
E-selectin	A selectin cell adhesion molecule expressed only on endothelial cells activated by cytokines. E-selectin has a cassette structure: an N-terminal, C-type lectin domain, an EGF (epidermal-growth-factor)-like domain, 6 Sushi domain (SCR repeat) units, a transmembrane domain (TM), and an intracellular cytoplasmic tail (cyto).	Facilitate leucocyte cell trafficking by recognising ligand surface proteins. Cancer cells secrete inflammatory cytokines, such as IL-1β or TNFα, to induce E-selectin at distant metastatic sites, enabling circulating tumour cells to get arrested at stimulated sites, roll along activated endothelium, extravasate, and cause metastases.	^197^
interferon	Belongs to cytokines and is typically divided into three classes: Type I IFN, Type II IFN, and Type III IFN.	Released by host cells to fight viral infections and regulate the immune system. IFN I acts as an anticancer agent via the antiproliferative/pro-apoptotic activity.	^198,199^
TNF	A pleiotropic cytokine consisting of various transmembrane proteins with a homologous TNF domain.	A master regulator of inflammation; exogenetic TNF exists the anticancer activity by destroying the vascular bed to induce haemorrhage. TNF made by malignant cells or/and host cells contribute to inflammation-associated cancer and cause genetic damage.	^200,201^
IL-6	The IL-6 family of cytokines consists of IL-6, IL-11, IL-27, IL-31, OSM, LIF, CNTF, CT-1, and CLCF1, which are defined by the usage of common b-receptor signalling subunits.	The pro-tumorigenic actions of IL-6 cytokine family members are elicited by both direct intrinsic effects on cancer cell activities (cell proliferation, survival, migration, invasion, and metastasis) and indirect effects on the stromal cell compartment, such as modulation of inflammation, immunosuppression, and angiogenesis, which shape the local tumour microenvironment.	^202^
NF-kB	A transcription factor family, sharing a Rel homology domain in the N-terminus.	A key participant in innate and adaptive immune responses. NF-kB is activated by inflammatory cytokines and activates the survival genes within cancer cells and inflammation-promoting genes in components of the tumour microenvironment.	^203,204^
Cytokines	A broad and loose category of small proteins (5–20 kDa). Cytokines include chemokines, interferons, interleukins, lymphokines, and tumour necrosis factors	Act as immunomodulating agents. Cytokines released in response to infection, inflammation, and immunity can inhibit tumour development and progression, whereas, host-derived cytokines promote cancer growth, attenuate apoptosis, and facilitate invasion and metastasis.	^205^
E-Cadherin	A calcium-dependent cell–cell adhesion glycoprotein, composed of five extracellular cadherin repeats, a transmembrane region, and a highly conserved cytoplasmic tail.	Controls cell–cell adhesion and maintains epithelial phenotype of cells. Loss of E-cadherin in cancer cells leads to the epithelial to mesenchymal transition (EMT) and causes a more migratory mesenchymal phenotype.	^161,206^
Vimentin	A type III intermediate filament (IF) protein	Vimentin plays a key role in supporting and anchoring the position of organelles in the cytosol. Vimentin is a marker for epithelial–mesenchymal transition (EMT) and promotes an aggressive phenotype of cancer cells.	^207^
Chemokines	A superfamily of small secreted molecules, defined by four invariant cysteines and categorised based on the sequence around the first two cysteines, thereby leading to two major and two minor subfamilies	Chemokines act as a chemoattractant to guide the migration of cells, including lymphocytes and cancer cells.	^94,179–199^
Platelets	A component of blood	Induce CTC-platelet aggregates, enhance thrombopoiesis; reciprocally, platelets reinforce CTC growth with proliferation signals, antiapoptotic effect, and angiogenic factors. Platelets activate tumour invasion and sustain metastasis by inducing an invasive EMT phenotype of CTCs, promoting CTCs’ survival in circulation, arrest in the endothelium, and extravasation; protects CTCs from immune attack.	^194,208^

## Key factors that spark CTC adhesion

Successful heterocellular adhesion of CTCs to endothelial cells in distant tissues requires an extensive variety of ligands and receptors participating in the process, including VCAM/ICAM, platelets, selectins, integrins, CD44, and cadherins. The initial slight adhesion could cause subsequent stable adhesion via a chain of reactions among the adhesion-related ligands and receptors in the microenvironment.^128,129^

### Integrins and metastatic adhesion

Integrins are important transmembrane heterodimers consisting of 18 α-subunits and 8 β-subunits, which interact with extracellular matrix (ECM) ligands.^130^ Integrins play a key role in metastasis.^131^ Several integrins in CTCs are involved in their adhesion to the endothelium, transmigration across it for extravasation, and for following adhesion to the stromal matrix.^132,133^ Integrins in CTCs mediate the attachment of CTCs to circulating cells for their extravasation. For example, integrin-αVβ3 in CTCs are needed for their attachment to platelets under the condition of flow.^134,135^ Integrin-β1, integrin-β4, and integrin-αVβ3 contribute to the strong adhesion between CTCs and endothelial cells in vitro. Both β1 and αVβ3 integrins appear to be constitutively active, and do not need any chemokine for their activation.^136^ The α6β4 integrin is essential for the firm adhesion of CTCs to endothelial cells following the initial binding of E-selectin to the CTCs.^137^ Integrinβ1 deletion in cancer cells has been shown to decrease the interaction between cancer cells and endothelial cells in vitro.^138^ Although integrins are clearly involved in cancer cell extravasation, the function of α-integrin in this regard has not been elucidated yet. Ginsenoside Ro (a chemical separated from the abortion-related herb *Achyranthes bidentata Blume*) had shown potent inhibition of metastatic dissemination of colon CTCs, including their adhesion and invasion to human endothelium, by inhibiting integrin αvβ6.^112^ Nude mice were pre-treated with oral ginsenoside Ro, and then i.v. injected with colon cancer cells, observably prevented lung metastasis with downregulation of integrin αvβ6 without obvious toxicity.

### VCAM/ICAM and metastatic adhesion

Vascular cell adhesion molecule (VCAM) and intercellular adhesion molecule (ICAM) belong to the Ig superfamily.^139^ ICAM-1 is the main ligand for β2-integrins. It normally expresses at basal levels in several cell types, including endothelial cells, keratinocytes, fibroblasts, and leucocytes. ICAM expression increases upon inflammation caused by IFN-γ, TNF-α, and IL-1β.^140^ In inflammatory tissues, ICAM-1 expressed on endothelial cells binds to β2-integrins LFA-1 and Mac-1 in CTCs, thereby facilitating their transendothelial migration to the inflammation site. ICAM-1 up-regulation is associated with various autoimmune, inflammatory, and allergic diseases, along with cancer metastasis.^141^ Transmembrane protein VCAM-1 is mainly expressed on endothelial cells and it can be activated by any kind of the extracellular stimuli, including pro-inflammatory cytokines and reactive oxygen species, such as TNFα and interleukin-1. Transmembrane VCAM-1 is composed of an extracellular field containing 7 homologous immunoglobulin (Ig)-like fields, a transmembrane field, and a cytosolic field. During inflammation, VCAM-1 binds directly to integrins on CTCs within the extracellular domain.^142^ It is critical for recruiting inflammatory factors to participate in this molecular interaction.

### Platelets and metastatic adhesion

An appropriate microenvironment can facilitate the activated CTCs to trigger the adhesion-invasion-extravasation metastatic cascade in their pre-metastatic niches. Platelet is the first type of cells to reract with CTCs. After the CTCs enter the bloodstream, they gather around the CTCs almost immediately.^143^ Platelet-CTC interaction is conducive to the progression of CTC-induced metastasis by preserving CTCs from shear stress and immune attack from natural killer cells, thereby helping CTCs to be entrapped in the capillary bed and prepare for extravasation. The activated platelets can cloak CTCs, resulting in CTCs exhibiting the platelet biomarkers CD61 and P-selectin. Platelet-derived, rather than CTC-derived, signals have been shown to be required for the recruitment of granulocytes to CTCs to form early metastatic niches, probably within minutes.^144^ Upon contact with CTCs, platelets cause the secretion of CXCL5 and CXCL7 chemokines, resulting in granulocyte recruitment. Blockade of the CXCL5/7 receptor CXCR2, or transient depletion of either platelets or granulocytes prevents the formation of early metastatic niches and significantly decreases metastatic seeding and progression. CTCs have been shown to boost platelet coagulation by expressing the thrombin and releasing the microparticles, and the resulting coagulation further increased cancer metastasis in reported models.^145^ On the contrary, the inhibition or depletion of platelet can inhibit metastasis in mouse models.^146,147^ For example, the multifunctional NO-carrying molecule S-nitrosocaptopril can act on both CTCs and platelets to interrupt platelet/CTCs interplay and adhesion to endothelium, thus suppressing CTC-based pulmonary metastasis in vivo.^148^ Platelets facilitate extravasation of CTCs by raising the attachment of CTCs to endothelial cells.^133^ Platelet-derived P-selectin expedites both liver and lung metastases.^149^ Moreover, platelets release TGFβ and ATP to promote metastatic seeding in the lung, CTC extravasation, and metastasis.^150–152^ Platelets obviously strengthen the lung metastasis of melanoma but do not affect the liver metastasis.^149^ When adhered to endothelial cells, platelets can recruit leucocytes and stimulate their integrins via P-selectin mechanism.^153–155^

### Selectins and metastatic adhesion

Selectins are important receptors for heterocellular adhesions. The single-chain transmembrane glycoproteins include E-selectin (in endothelial cells), L-selectin (in leucocytes), and P-selectin (in platelets and endothelial cells). They mediate cell tethering and rolling interactions through recognition of carbohydrate ligands on cancer cells to enhance distant organ metastases.^156,157^ The tetrasaccharide sialyl Lewis X (sLe^x^) antigen or its isomer sialyl Lewis a (sLe^a^) are selectin ligands containing glycoproteins and glycolipids.^158^ Different glycoprotein ligands for endothelial selectin (E-selectin) are expressed in CTCs, and we have used sLe^x^-modified and sLe^a^-modified glycoproteins in colon CTCs as CTC biomarkers.^85^ Interaction of CTCs with E-selectin is necessary for metastases.^159,160^ Usually E-selectin is not expressed in inactive endothelial cells, but can be induced by inflammatory factors, such as tumour necrosis factor-α (TNF-α), NF-κB, and cytokines. E-selectin only participates in the interaction of CTCs with endothelial cells after amplification or stimulation of macrophages. It may not participate in the primary capture of CTCs on the endothelium in vivo. In mouse lungs, the conditioned medium from cancer cells resulted in E-selectin-upregulated foci in endothelial cells, correlating well with sites of CTCs attached in the lungs after 5-h injection of CTCs.^156^ However, if E-selectin is related in the early CTC adhesion or if it conductive to a later enhancing of the reaction of CTCs with endothelial cells would need further investigation.

### Cadherin and metastatic adhesion

Cadherin includes E-cadherin and N-cadherin (Neuronal cadherin; or cadherin 2), which are expressed by endothelial cells, as well as by many cancer cells.^161^ E-cadherin is a calcium-dependent cell–cell adhesion glycoprotein that controls cell–cell adhesion and maintains epithelial phenotype of cells. N-cadherin is a typical mesenchymal marker. We used a bioactive polysaccharide separated from a traditional Chinese medicinal herb *A. bidentata* as a single cancer metastasis chemopreventive agent, and found that the polysaccharide can prevent cancer metastasis safely and effectively in both in vitro and in vivo settings by increasing the expression of E-cadherin and β-catenin and decreasing that of N-cadherin, vimentin, and Snail.^114^ Since the polysaccharide produced a dose-dependent chemoprevention of CTC-induced metastasis in mouse lungs, the chemoprevention was believed to be, in part, related to the regulation of both E-cadherin and N-cadherin. Moreover, the expression of N-cadherin in breast cancer cells was demonstrated to motivate metastasis,^162^ and the expression of N-cadherin in mammary epithelium of breast cancer mouse model promoted pulmonary metastasis.^163^

### Chemokines and extravasation

Chemokines and its 7 transmembrane-spanning G protein-coupled receptors play key roles in cancer metastasis. The metastatic organotropism can be explained by the type of complementary chemokines and their receptors expressed in the target endothelium of vasculature and CTCs.^164^ Among all chemokines, CXC-chemokine ligand 12 (CXCL12) has been well investigated in extravasation and metastasis of cancer cells.^165^ CXCL12 released by stromal cells at the distant tissues can draw tumour cells expressing CXCR4 and CXCR7 to activate extravasation, migration, and adhesion of cancer cell. Abortifacient metapristone (RU486 derivative) has been shown to interrupt the CXCL12/CXCR4 axis, resulting in ovarian metastatic chemoprevention. Metapristone, at low concentrations (<IC_50_) significantly reduced CXCL12-induced CXCR4 expression in ovarian cancer cells, and down-regulated the CXCR4-related mRNAs and intracellular proteins by interfering the CXCL12-activated Akt and ERK signalling pathways.^108^ Metapristone also inhibited the events of cellular invasion, migration, and adhesion. The inhibition is related to downregulation of the invasive molecules MMP-2, MMP-9, COX-2, and VEGF without affecting the adhesion molecules ICAM-1, integrins α1, α3, α5, α6 and β1. CXCL12 activation increased cancer cells adhesion to endothelial cells.^166–169^ The devotion of CXCR4 and CXCR7 to CXCL12 responses may rely on the cell type. The expression of exogenous CXCR4 enhanced melanoma cells adhesion to endothelial cells in vitro and the lung metastasis in vivo,^170^ however, the CXCR7 inhibitors decreased TEM of lymphoma cells in vitro.^171,172^ Chemokine production by CTCs can promote extravasation. For example, CC-chemokine ligand 2 (CCL2), which is produced by colon cancer cells and breast cancer cells, interacts with CC-chemokine receptor 2 (CCR2) in endothelial cells and/or in myeloid cells to increase extravasation-related metastasis in animal lungs.^173,174^ Chemokine production by CTCs can also draw leucocytes to promote the CTCs’ avoid of immune system and resulting extravasation in various malignancies.^175–177^

### CD44 and metastatic adhesion

CD44 expression in CTCs strongly correlates with their adhesion to endothelial cells and with colorectal cancer metastasis.^73,178^ The specific glycosylated phenotypes of CD44 can react with selectins. CD44 is necessary for the adhesion of breast, colorectal, prostate CTCs to endothelial cells and for TEM.^179,180^ Human growth factor raises the expression of CD44 in breast CTCs and enhances their adhesion to bone marrow cells as well as to transendothelial membrane.^179,181,182^ All colorectal CTCs are reported to express CD47, with only <61.2% being CD44 positive.^73^ CD44 binding induces signalling that results in changes in CTC gene expression, thereby contributing to transendothelial membrane;^178^ for example, CD44 crosslinking in colorectal and breast CTCs induces the expression of α4β1 and αLβ2 integrins, thereby resulting in adhesion between CTCs and endothelial cells and subsequently to transendothelial membrane.^183,184^

## Cell surfactant concept and practices

Anti-adhesive agents (termed here as “cell surfactants” or “cancer metastasis chemopreventive agents”) constitute a group of substances with pharmacological activity that can specifically inhibit the contact/adhesion process between heterogeneous cells induced by various bioactive substances (such as inflammatory factors, adhesion molecules, platelets, etc.) in cell microenvironment (Table 2). These substances do not affect the normal biological activity of different cells within the effective concentration range of the substance. Contrary to cell surfactants, chemical surfactants can produce wetting, emulsification, solubilisation, and foaming to significantly reduce the surface tension of liquids. Stearic acid wax, cationic surfactant, and amphoteric surfactant are commercial chemical surfactants, which are generally composed of hydrophilic and lipophilic groups. The classification of chemical surfactants is mainly defined by the hydrophilic groups contained in them. Cell surfactants, however, have no clear hydrophilic and lipophilic groups, and their mechanism of action is not realised by changing the interfacial properties and reducing the surface tension between dirt, mould, and adsorption surface. Instead, the mechanism of cell surfactants is to inhibit the contact/adhesion between heterogeneous cells that is triggered by inflammatory factors, adhesion molecules, and platelets in the cell microenvironment.

The concept of anti-adhesion therapy had previously been tested for the prevention of bacterial adhesion to targeted tissues, thereby blocking infection.^117^ Just like chemical surfactants that change the interfacial properties and reduce the surface tension between dirt or moulds and their adsorption surface, resulting in no mould colony forming on the surface, the anti-adhesion strategy using carbohydrates, glycodendrimers, and glycopolymers interferes with pathogen attachment, thereby preventing pathogen adhesion and the subsequent infection.^185^ The non-adhering anoikis bacteria subsequently perish owing to loss of their supporting basis. The anti-adhesion therapy is a safe, feasible, and operative strategy. For instants, human breast milk that contains many oligosaccharides acts as anti-adhesives.^186,187^ In addition, intervention at the starting step of infection or cancer metastases is a conceptually highly attractive alternative to conventional antibiotics or anticancer drugs that face a serious challenge of drug-induced resistance. Below are some successful examples of tested cancer metastasis chemoprevention.

### Embryo and CTC adhesion

Global epidemiological studies have concluded that cancer deaths are lower after long-term use of contraceptives.^188^ Inspired by these global epidemiological results, we hypothesised that if an abortifacient could interfere with implantation of the fertilised egg (blastocyst) into endometrium, and lead to infertility,^189^ then it may also interfere with the implantation of CTCs into endothelium (Fig. 3); therefore, abortifacients may be a good class of cancer metastasis chemopreventives to prevent cancer metastatic cascade from getting initiated.

Blastocyst implantation is a complex process including attachment of the blastocyst to the receptive endometrium and invasion of the trophoblastic cells of the conceptus into the endometrium and basement membrane.^189^ After analysing the molecular and cellular similarities and differences between embryonic implantation to uterine endometrium and breast CTC adhesion to vascular endothelium,^190^ we found that many biological factors were shared by both the embryonic implantation system and CTC adhesion-invasion system, including integrins, cellular adhesion molecules (CAM), such as EpCAM, ICAM, VCAM, and selectin, hormone receptors, sialyl Lewis X and MMPs.^112^ The in vivo inhibition by abortifacients of both embryo implantation to uterus and CTC metastasis to lung further revealed similarities in molecular and cellular embedding between the reproduction system and cancer metastases, and provided fundamentals for abortifacients to intervene CTC adhesion/invasion to the distant metastatic organs in vivo.^112,113^

Metapristone, the most active metabolite of mifepristone (RU486), is a good candidate for cancer metastatic chemoprevention, owing to its good anti-adhesion, in vivo biostability, and few side effects. RU486 is a blockbuster abortifacient,^191^ and its mechanisms of abortion include antiprogesterone and anti-glucocorticoid interfering heterotypic cell adhesion to basement membrane, and inhibiting human sperms from binding onto the zona pellucida mediated by the sialyl-Lewis(x) oligosaccharide.^192^ Recent studies have revealed its ability to inhibit cell migration and growth of various cancer cell lines by downregulating CDK2, BCL-2 and NF-kB. RU486 has been used in clinical trials in the USA as an anticancer drug for breast tumour, meningioma, gliomas in the central nervous system, prostate cancer, ovarian and endometrial cancer, and gastric adenocarcinoma.^193^ However, the anticancer trials have been unsuccessful owing to the difficulty in reversing cancer deaths after metastases.

We synthesised N-monodemethyl mifepristone, i.e., metapristone, in large scale, and characterised its physicochemical and pharmacological properties.^105^ Metapristone showed a cytostatic effect on cancer cells. It blocked cancer cells at G0 phase and apoptosized them. Metapristone interrupted adhesion of colorectal, breast, ovarian, lung, and melanoma cancer cells to endothelial cells of distant metastasis organ intima.^105–109^ Pharmacoproteomic analysis uncovered that metapristone intervened the epithelial marker E-cadherin and mesenchymal marker vimentin in breast cancer cells, and built the basis for cancer metastasis chemoprevention.^110^ Systems pharmacology analysis suggested that metapristone mediated the FAK-Src-Paxillin complex to interfere with heterocellular adhesion. Metapristone had been shown to interrupt cytokine CXCL12/CXCR4 axis, resulting in ovarian metastatic chemoprevention.^106^

### Anti-adhesive medicine for abortion

The traditional Chinese medicine (TCM) *Murraya paniculata* (L.) is associated with abortion, anticoagulation, anti-inflammation, and antimicrobial and anticancer effects. To exploit safe and effective cancer chemopreventive agents from the huge botanical medicine resource, we extracted the effective component G from *M. paniculata*, since it contains the flavonoids glycoside and coumarins. To quantitatively distinguish cancer metastasis chemopreventive agents from cytotoxic anticancer drugs, we created the adhesion/inhibition ratio (AIR), to define and screen the cancer metastasis chemopreventive agents. The AIR is calculated by dividing IC_10_ (the mean drug concentration causing a 10% growth inhibition of cells) by EC_50_ (the mean drug concentration causing a 50% inhibition of the hetero-adhesion between cancer cells and endothelial cells).^37^ The larger the AIR, the more likely does the drug function as a cancer metastasis chemopreventive agent by primarily inhibiting hetero-adhesion, rather than as a cytotoxic drug that inhibits hetero-adhesion by cell killing, with possible side effects.

The component G revealed the ability to scavenge free radicals, and restrained embryo implantation into human endometrium and HT29 cell implantation into human endothelium without obvious cytotoxicity, proved by the significant adhesion inhibition effect. By downregulating integrins and CD44 of cancer cells, as well as E-selectin of endothelial cells, component G inhibited invasion and migration of cancer cells. In CTC-induced lung metastasis animal model, the component G had obvious lung metastasis inhibition without remarkable side effects.

We isolated and characterised 5 coumarin-containing components (Z1–Z5) from the extract of *Murraya paniculata*. These components and their mixture down-regulated EpCAM expression and inhibited the adhesion of cancer cells to human endothelial cells. Rat coagulation study demonstrated that warfarin and coumarin mixture from the botanical extracts prolonged prothrombin time, indicating important roles of both platelets and cell adhesion molecules.

With further exploitation of the cancer pre-metastatic chemopreventive agents, another herbal medicine drew our attention.^112^ The medicinal herb *Achyranthes bidentata* (*A. bidentata*) can be found all over Asia, including India, Korea, Japan, and China. Its role in abortion had been recorded 2700 years ago (505–557 A.D.). Many active components were found in the herb, including phytosterone and phytoecdysteroids, saccharides, saponins, and others. We separated five compounds from the herb root, among which, ginsenoside Ro was the most potent in inhibiting embryonic implantation within non-cytotoxic concentrations. Ginsenoside Ro specifically inhibited the metastatic dissemination capability of colon CTCs, including their adhesion/invasion to human endothelium as well as migration by inhibiting integrin αvβ6, matrix metalloproteinase-2/9 (MMP-2/9), and ERK phosphorylation via HT29. Forty-day oral administration of ginsenoside Ro significantly prevented CTC-induced lung metastasis with downregulation of integrin αvβ6 without significant toxicity. Further, we isolated a polysaccharide from the roots of *A. bidentata*. The polysaccharide bound to the extracellular domain of epidermal growth factor receptor (EGFR), inhibited its activation and downstream signalling pathways, blocked EMT process, and inhibited expression of Snail, N-cadherin and MMP-2/9, but upregulated E-cadherin.^114^ In LLC murine artificial pulmonary metastatic model, where subcutaneously implanted tumours were surgically removed and the resulting lung metastasis was caused by blood CTCs, the polysaccharide significantly reduced CTC-induced lung metastasis. These studies suggested that these abortion-related botanical medicines may be used for CTC-related cancer pre-metastasis chemoprevention.

### Safe/active prevention combination

**Fig. 5:**
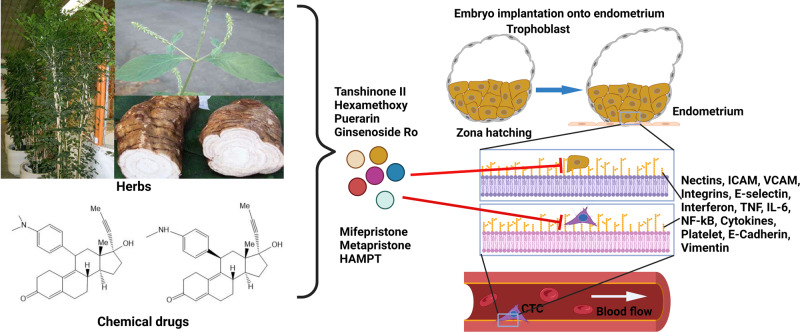
Safe and effective metastasis chemopreventive agents developed. Based on similarities in adhesion-embedding between embryos and CTCs, abortifacients mifepristone and metapristone are repurposed and developed for cancer metastasis chemoprevention. Components extracted from abortion botanic medicines are Hexamethoxy (from abortion botanic plant Murraya paniculata) Ginsenoside R0 (from abortion herb Achyranthes bidentata). Puerarin and Tanshinone II are chemicals obtained from traditional cardiovascular herbs Pueraria lobata and Salvia miltiorrhiza, respectively. HAMPT (abbreviated for Highly Active Metastasis Preventing Therapy) is a quardruple-combined drug that targets various factors in the distant niche. Created with biorender

Based on CTCs’ disseminating organotropism and their niches, we created a quadruple combination drug named HAMPT (abbreviated for Highly Active Metastasis Preventing Therapy), which not only targeted various factors in the distant niche (Fig. 5), but also enhanced the patient’s own recovery power to suppress metastatic potential.^37^ HAMPT is composed of aspirin, lysine, mifepristone and doxycycline. Inhibition of cancer cell adhesion by HAMPT, a pharmaceutical formulation, is mediated by downregulating cell adhesion molecules, such as CAM and integrins. CTCs were inhibited by HAMPT through the activation of platelets,^144^ and hence interfered with the adhesion and invasion of CTCs to the underlying stroma. Intervention of platelets’ crosstalk with CTCs prevented the early formation of metastatic niches and significantly reduced metastatic seeding.^194^ Cancer cells were dormant after HAMPT treatment. Thirty-day oral administration of HAMPT (33.5–134 mg/kg) to CTC-injected mice got obvious reduction of metastasis, without remarkable side effects, in a dose-dependent manner. There was no significant toxic response in rats when HAMPT was given at a dose 20-fold greater than its therapeutic dose for 50 days. This paradigm-shifting study provided the strong proof that metastasis can be restrained by using affordable old drugs to prevent CTCs from germinating on the metastatic niche without the need of cytotoxicity. The novel strategy might revolutionise the future of cancer research and treatment.

### Outlook

In this review, we proposed, for the first time, that intervention of pathogenic adhesion can prevent and control the occurrence and development of some serious diseases that are not easy to treat otherwise. As shown in Fig. 3c, there are fundamental differences between the decades-old cancer chemoprevention and the new cancer metastasis chemoprevention, the latter focusing on preventing CTCs from pathogenic adhesion to capillary endothelium of distant metastasis tissues. Just as chemical surfactants prevent pollution by inhibiting the adhesion of dirt and mould on clean surfaces, cell surfactants can inhibit the inappropriate adhesion of various bioactive substances (CTCs, viruses, monocytes, platelets, etc.) to an unsuitable tissue microenvironment, thereby preventing potential diseases from getting triggered. Incorrect heterocellular adhesion can become pathogenic, resulting in various serious diseases that cannot be completely cured later. For example, the adhesion of CTCs to the endothelial cell layer of metastatic tissue may lead to cancer metastases; the adhesion of coronavirus to the alveolar surface may lead to respiratory failure; and the adhesion of monocytes to the endothelial cell layer of coronary artery may lead to atherosclerotic nucleus. The cell-adhesion molecules can be activated mainly by inflammatory factors, such as TNF, NF-κB, cytokines, chemokines and platelets, and therefore, the cell surfactants or anti-adhesion drugs (Fig. 5) should have great potential in inhibiting inflammation and platelet aggregation. There are many such drugs that can be repurposed for new applications in a relatively safe manner. The introduction of anti-adhesives or cell surfactants into cancer pre-metastasis chemoprevention has demonstrated a safe, effective, and affordable strategy, which may, in turn, open a new horizon in the war on cancer to significantly reduce the death and suffering of patients with metastatic cancer in future.

## Supplementary information

RightsLink Printable License
